# Ultraconformable Integrated Wireless Charging Micro-Supercapacitor Skin

**DOI:** 10.1007/s40820-024-01352-1

**Published:** 2024-02-19

**Authors:** Chang Gao, Qing You, Jiancheng Huang, Jingye Sun, Xuan Yao, Mingqiang Zhu, Yang Zhao, Tao Deng

**Affiliations:** 1 School of Electronic and Information Engineering, Beijing Jiaotong University, Beijing, 100044 People’s Republic of China; 2 School of Microelectronics, Tianjin University, Tianjin, 300072 People’s Republic of China; 3 Key Laboratory of Cluster Science Ministry of Education of China, Beijing Key Laboratory of Photoelectronic/Electrophotonic Conversion Materials, School of Chemistry and Chemical Engineering, Beijing Institute of Technology, Beijing, 100081 People’s Republic of China

**Keywords:** Micro-supercapacitor, Electronic skin, Supercapacitor skin, Wireless charging energy storage device

## Abstract

Conformable and wireless charging energy storage devices play important roles in enabling the fast development of wearable, non-contact soft electronics. However, current wireless charging power sources are still restricted by limited flexural angles and fragile connection of components, resulting in the failure expression of performance and constraining their further applications in health monitoring wearables and moveable artificial limbs. Herein, we present an ultracompatible skin-like integrated wireless charging micro-supercapacitor, which building blocks (including electrolyte, electrode and substrate) are all evaporated by liquid precursor. Owing to the infiltration and permeation of the liquid, each part of the integrated device attached firmly with each other, forming a compact and all-in-one configuration. In addition, benefitting from the controllable volume of electrode solution precursor, the electrode thickness is easily regulated varying from 11.7 to 112.5 μm. This prepared thin IWC-MSC skin can fit well with curving human body, and could be wireless charged to store electricity into high capacitive micro-supercapacitors (11.39 F cm^−3^) of the integrated device. We believe this work will shed light on the construction of skin-attachable electronics and irregular sensing microrobots.

**Supplementary Information:**

The online version contains supplementary material available at 10.1007/s40820-024-01352-1.

## Highlights

- An ultraconformable skin-like integrated wireless charging micro-supercapacitor (IWC-MSC) could be wireless charged to store electricity into high capacitive micro-supercapacitors (11.39 F cm^−3^), and fits well with human surface.
- Building blocks of IWC-MSC skin are all evaporated by liquid precursor, and each part of the device attached firmly benefitting from the liquid permeation, forming a compact and all-in-one configuration.
- The electrode thickness easily regulated varying from 11.7 to 112.5 μm by controlling the volume of electrode solution precursor.

**Supplementary Information:**

The online version contains supplementary material available at 10.1007/s40820-024-01352-1.

## Graphical abstract

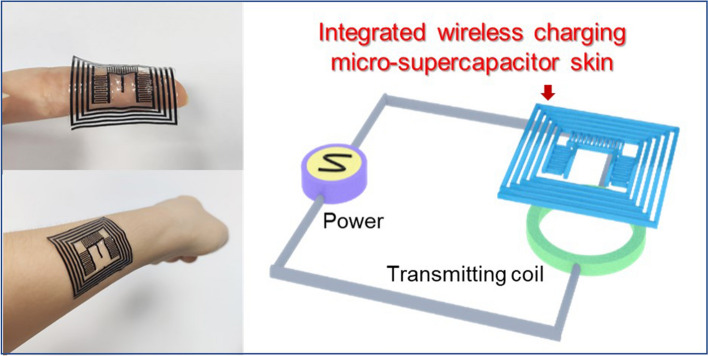

## Introduction

Rapid development of the Internet of things (IoTs) stimulates demands for microelectronic devices in the fields of wearables, electronic skin, digitalization of health and implantable electronics [1–3]. For such a purpose, energy storage device is indispensable to supply electricity, and required to be flexible, thin and lightweight to conform easily onto the skin. Micro-supercapacitor (MSC) is one of the promising micro energy storage devices, featuring with fast charging and discharging rate, long cycling life and high power density [4–7], which has a great potential to be used as energy supplying device in microelectronics. In practical, MSC requires periodically charging of external power source by connecting wires, meaning additional manpower [3]. Besides, the charging equipment is usually bulky, easily damaging the MSC by physical electrode contact. Therefore, it is imperative for MSCs featuring with adaptive merits, such as wireless charging, to replenish timely and sufficient electricity without wired external connections for power supply.

Many investigations assemble MSC with wireless charging coil to pursue an integrated wireless charging device [8]. For example, Shen [9] fabricated an all MXene-based seamlessly integrated system with wireless charging coil, micro-supercapacitor and photodetector, and used wireless charged MSC to drive the surface-modified (dodecyl triethoxysilane) DCTES-MXene-based photodetector. Mousavi [10] built an integrated wireless charging, energy storage and sensing system by laser-scribed graphene, containing near-field communication (NFC) antenna, micro-supercapacitor, humidity sensor and resistor. Our previous work [11] combined wireless charging coil and micro-supercapacitor together by using graphite/activated carbon materials. Although most of the previous reports are devoted to build integrated structural and material wireless charging devices, it is still a challenge to build a satisfying skin-conformable integrated wireless charging micro-supercapacitor, which is skin-like to accommodate the local details of the target uneven surface as well as aesthetics [12]. The main reason is that the constructing materials are only flexible within limited area or angles, presenting flexural rigidity to some extent. In addition, the contacts among the substrate, wireless charging coil, supercapacitor electrode and electrolyte of these integrated devices are weak, causing that the integrated devices tend to crinkle and take apart during human motions. These problems severely constrain the application of IWC-MSC as a skin-like power source in human–machine interfaces, virtual reality/augmented reality platform and artificial robots [13–15].

In this work, we fabricated an ultraconformable and thin integrated wireless charging micro-supercapacitor (IWC-MSC) skin by evaporating solution mixture. All parts of the IWC-MSC skin are originated from solution precursors: the MSC electrodes and wireless charging coil are from poly(3,4-ethylenedioxythiophene) poly(styrenesulfonate) (PEDOT:PSS)/ionic liquid mixture solution; the substrate is from polyvinylidene fluoride–hexafluoropropylene copolymer (PVDF-HFP)/acetone solution precursor; the MSC electrolyte is from PVDF-HFP/acetone/ionic liquid solution mixture. By evaporating these curable solutions, each part was tightly attached with each other by dissolution, infiltration and permeation, forming an all-in-one configuration of IWC-MSC. In addition, since the supercapacitor electrodes and wireless charging coil are made by PEDOT:PSS and ionic liquid conductive solution, the IWC-MSC electrode thickness can be controlled by adjusting the solution volume facilely, varying from 11.7–112.5 μm. The prepared IWC-MSC, featuring with thin, ultraconformable and all-in-one configuration, fits well with the human surface and could be wireless charged to store electricity into micro-supercapacitors of the integrated device. Moreover, the MSC of the integrated device shows a good volumetric capacitance of 11.39 F cm^−3^, which is higher than most of PEDOT-based micro-supercapacitors. We hope that this all-in-one conformable IWC-MSC skin will be applied to the future electronic skin, microrobots and irregular human organs sensing systems.

## Experimental Section

### Materials

Poly(vinylidene fluoride-co-hexafluoropropylene) (PVDF-HFP), poly(3,4-ethylenedioxythiophene) poly(styrenesulfonate) (PEDOT:PSS), acetone and 1-ethyl-3-methylimidazolium bis(trifluoromethylsulfonyl) imide ([EMIM][TFSI]) are purchased from Sigma-Aldrich.

### Experiment

#### Substrate Precursor Preparation

4 g poly(vinylidene fluoride-co-hexafluoropropylene) (PVDF-HFP) was dissolved into 40 mL acetone, and stirred for 3 h to obtain a homogeneous transparent substrate precursor in the room temperature.

#### Electrode Precursor Preparation

150 μL [EMIM][TFSI] ionic liquid and 6 mL PEDOT:PSS were mixed together and stirred vigorously for 10 min. This solution precursor needs to quickly drop on the substrate in the fabrication process. Otherwise, the ionic liquid would interact with PEDOT:PSS in a few minutes to form a hydrogel.

#### Electrolyte Precursor Preparation

1.5 g poly(vinylidene fluoride-co-hexafluoropropylene) (PVDF-HFP) was dissolved into 15 mL acetone, and stirred for 3 h. Then, 2 mL [EMIM][TFSI] ionic liquid was added into the mixture and stirred for 2 h to get a transparent electrolyte precursor in the room temperature.

#### Fabrication Process of IWC-MSC

Firstly, 3 mL PVDF-HFP/acetone substrate solution precursor was dropped on the glass plate and dispersed by a scraper, where the liquid precursor thickness was controlled by the scraper within 300 μm. The solution was quickly evaporated in 10 min at room temperature, leaving a PVDF-HFP thin and transparent substrate film on the glass. Then, 4 mL PEDOT:PSS/[EMIM][TFSI] electrode liquid precursor was dropped on the PVDF-HFP substrate, and dispersed by scraper in the same way. The volume of electrode solution could be adjusted by different types of scrappers (from 50 to 400 μm thick) according to the thickness of the electrode requirement. After dropping the electrode liquid precursor, the glass with substrate film and electrode liquid was placed on the heating platform, and heated at 50 °C for 20 min to form a stationary electrode gel. This precedure is to secure the flowable electrode liquid precursor. Then, heating the above glass plate at 120 °C for 3 min in the air to make the electrode dry. This dried electrode is a high conductive layer (called PE layer), which was the original material simultaneously used as MSC electrodes and wireless charging coil. If the electrode gel is still wet, in which PEDOT:PSS solution contains much water, heat the glass plate for a longer time. The surplus ionic liquid can be washed away by distilled water and dried in the air at room temperature. Subsequently, using the laser to scribe the conductive PE layer into the designed IWC-MSC pattern. The prepared device now contains MSC electrodes and wireless charging coil. To form MSC solid electrolyte, the electrolyte solution precursor was dropped on the surface of MSC electrodes, and evaporated in the air at room temperature for 20 min to obtain an all-solid-state IWC-MSC device. The prepared IWC-MSC could be scrapped from glass plate by a spade facilely, which presented a soft and thin appearance.

### Electrochemical Measurements

Electrochemical tests were carried out by the two-electrode system of electrochemical workstation. Cyclic voltammetry, galvanostatic charging and discharging tests, and electrochemical impedance spectroscopy (EIS) were measured by electrochemical workstation CHI 760E Instruments Inc. Shanghai, China). Calculation of areal capacitance, energy density and power density consulted with our previous papers [16].

Volumetric capacitance *C*_v_ was obtained as following:

1 $$C_{{\text{v}}} \left( {{\text{F}}\,{\text{cm}}^{ - 3} } \right) = \frac{I \left( A \right) \times \Delta t \left( s \right)}{{\Delta V \left( V \right) \times V\left( {{\text{cm}}^{{3}} } \right)}}$$

where *C*_v_, *I*, Δ*t*, Δ*V* and *V* are the volumetric capacitance (F cm^−3^), charge/discharge current (A), discharge time (s), discharge voltage (V) and the volume of the electrode (cm^3^), respectively.

The energy density and power density were calculated using the following equations [17]:

2 $$E\, \left( {{\text{Wh}}\,{\text{cm}}^{ - 3} } \right) = \frac{{C_{{\text{a}}} \left( {{\text{F}}\,{\text{cm}}^{ - 3} } \right) \times \Delta V^{2} \left( V \right)}}{7200}$$

3 $$P\, \left( {{\text{W}}\,{\text{cm}}^{ - 3} } \right) = \frac{{E\,\left( {{\text{Wh}}\,{\text{cm}}^{ - 3} } \right)}}{\Delta t \left( s \right)} \times 3600$$

where *E* and* P* are the energy density (Wh cm^−3^) and power density (W cm^−3^), respectively.

EIS measurements were conducted ranging from 0.1 to 10^6^ Hz with an amplitude of 10 mV.

### Characterization Measurements

Scanning electron microscope (SEM) pictures were recorded by SUPRA 55. Raman spectrum were tested by RM 2000 Microscopic Confocal Raman Spectrometer (Renishaw PLC, England) with a 633 nm laser. Powder X-ray diffraction (XRD) patterns were performed on a Netherlands 1710 diffractometer with a Cu Kα irradiation source (*λ* = 1.54 Å). Fourier transform infrared (FTIR) spectra were measured by a Bruker spectrometer (Equinox 55/S) adopting KBr pellets.

## Results and Discussion

### Design and Fabrication Process of IWC-MSC Skin

**Fig. 1:**
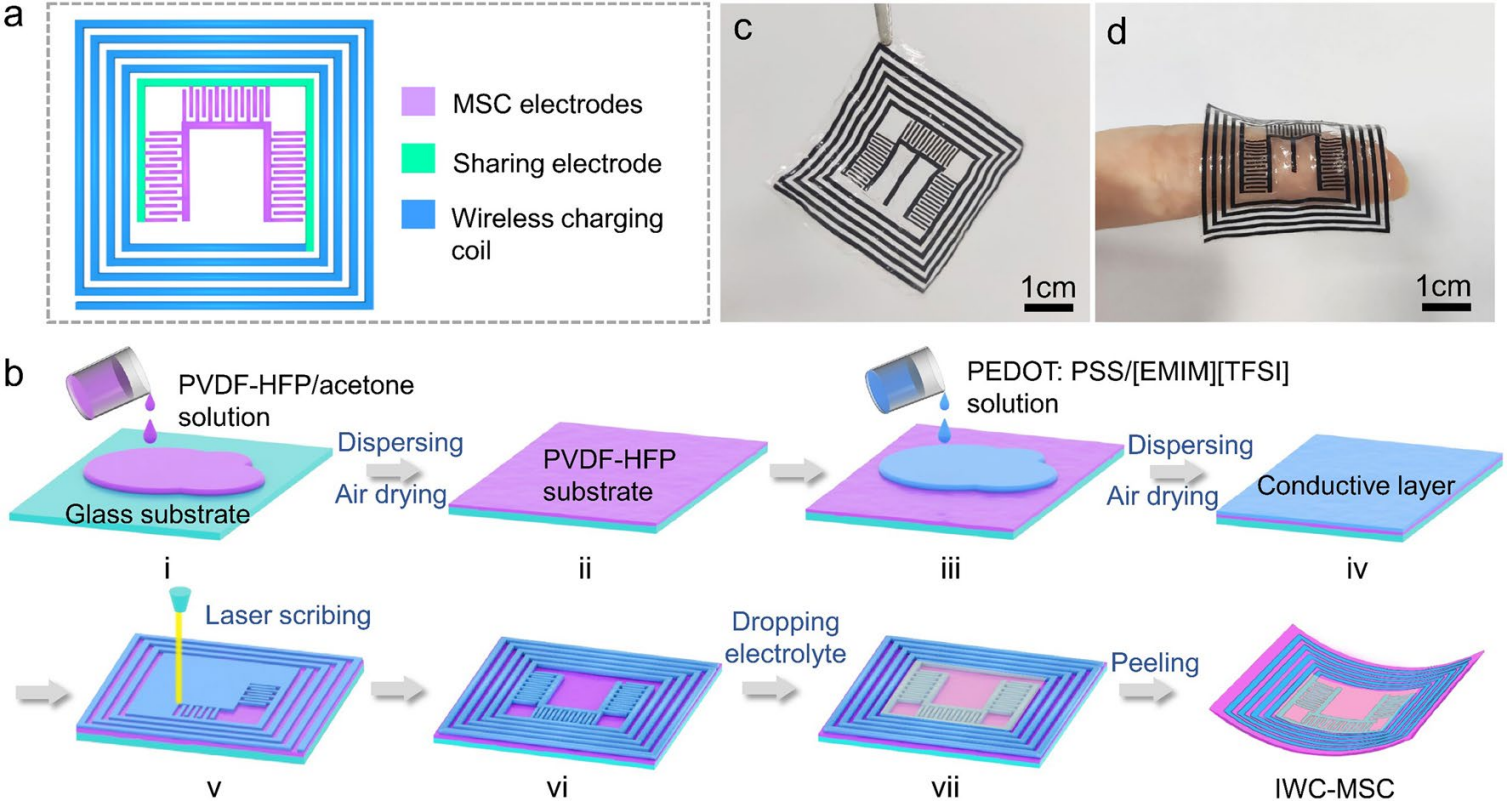
**a** A diagram of constructing mechanism of flexible wireless charging micro-supercapacitor integrated device, which is comprised of wireless charging coil and micro-supercapacitor electrodes in a seamless structure. **b** Fabrication process of IWC-MSC. **c**, **d** Pictures of the flexible and thin IWC-MSC

The integrated wireless charging micro-supercapacitor device is comprised of two parts: wireless charging coil (blue parts in Fig. 1a) and MSC electrodes (purple parts in Fig. 1a), where the MSC and wireless charging coil share the same green electrode to minimize the footprint of the integrated device [11].

The preparation process is shown in Fig. 1b. Before the experiment, PVDF-HFP was dissolved into acetone to make a precursor solution for IWC-MSC substrate. PEDOT:PSS was mixed with 1-ethyl-3-methylimidazolium bis(trifluoromethylsulfonyl) imide ([EMIM][TFSI]) ionic liquid to build a liquid precursor for the conductive layer of IWC-MSC. Firstly, PVDF-HFP/acetone solution was dropped on the glass plate (Fig. 1b-i) and dispersed by a scraper. The solution quickly evaporated in the air in few minutes, leaving a PVDF-HFP thin substrate film (Fig. 1b-ii). The substrate film is flexible, thin and transparent appearance, making it possible to build a robust structure (Fig. S1). Then, the PEDOT:PSS/[EMIM][TFSI] liquid precursor was dropped on the PVDF-HFP substrate (Fig. 1b-iii), and dispersed by the same way. It is confirmed that ion exchange of PSS^−^ of PEDOT:PSS to [TFSI]^−^ counterions of [EMIM][TFSI] ionic liquid helps PEDOT decouple from insulating PSS, greatly improving the conductivity from 260 kΩ cm^−1^ to 50 Ω cm^−1^ under electrostatic interaction [18, 19]. After air drying at 120 °C for 3 min, the high conductive layer with IWC-MSC was obtained (Fig. 1b-iv). Using the laser to scribe the conductive layer into the designed pattern of Fig. 1a. Only by few minutes, the electrodes of IWC-MSC were obtained (Fig. 1b-vi). The electrolyte precursor containing PVDF-HFP, [EMIM][TFSI] and acetone was dropped on the MSC electrodes, and evaporated in the air to obtain an all-solid-state MSC. Supported by the PVDF-HFP flexible substrate, the whole IWC-MSC device was easily peeled off from the glass plate. Notably, benefiting from the solution-processed substrate, electrodes (scribed from conductive layer) and electrolyte, the thickness of the IWC-MSC is under control. For convenience, only the thickness of the conductive layer, which transformed into MSC electrodes, was adjusted and discussed in the following parts. The fabricated IWC-MSC is displayed in Fig. 1c, d. It is obvious to see that the IWC-MSC is a thin, flexible, lightweight and all-solid-state film with a seamlessly integrated structure. The details of preparation process is shown in Experimental Section.

### Characterization of IWC-MSC Skin Structure and Materials

**Fig. 2:**
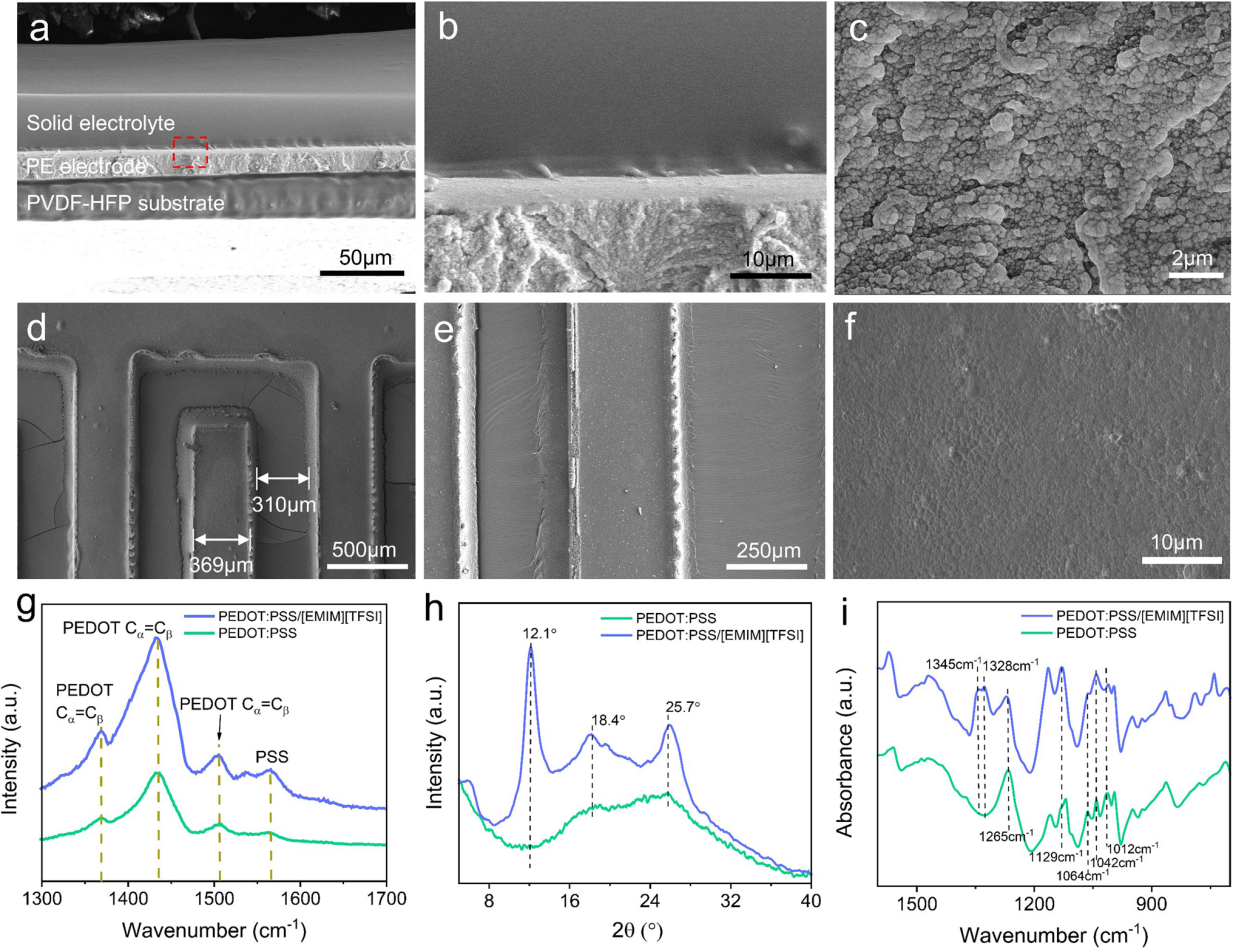
**a** SEM image of the cross section of MSC, including PVDF-HFP/[EMIM][TFSI] solid electrolyte, PE electrode and PVDF-HFP substrate. **b** Cross section interface between the solid electrolyte and PE electrode. **c** Magnified image of PE electrode. **d** SEM image of interdigital electrodes of MSC. **e** Magnified image of MSC electrode. **f** SEM image of the surface of MSC electrode. **g** Raman spectrum, **h** XRD pattern and **i** FTIR spectra of PEDOT:PSS and PE conductive layer

Structure of IWC-MSC was characterized by SEM. The cross-sectional view of IWC-MSC in Fig. 2a shows that the PEDOT:PSS/[EMIM][TFSI] (named as PE) electrodes, solid electrolyte and PVDF-HFP substrate are assembled together firmly, presenting as a completely all-in-one texture (Fig. 2a). The thickness of the solid electrolyte, PE electrodes and PVDF-HFP substrate are 32.1, 11.7 and 23.2 μm, respectively. It can be observed that the solid electrolyte is tightly attached with PE electrodes in Fig. 2b. This is because all three parts evaporated from liquid precursor, which tend to immerse and permeate each other to form an all-in-one robust structure. Besides, owing to the existence of [EMIM][TFSI] both in electrodes and electrolyte, it can be inferred that the aggregation of PEDOT:PSS and [EMIM][TFSI] via electrostatic interaction occurs both in the electrode and electrolyte, enhancing the binding force between them, which would deduce the ion transferring impedance crossing the electrode and electrolyte interface [20]. TEM pictures further reveal electrostatic interactions between [EMIM][TFSI] and PEDOT:PSS of the electrode, where PEDOT:PSS particles dispersed individually in Fig. S2a, but aggregated together in PEDOT:PSS/[EMIM][TFSI] in Fig. S2b. The magnified SEM image of PE electrode displays a compact and connective configuration (Fig. 2c), meaning that the PEDOT:PSS and [EMIM][TFSI] are homogeneously mixed and closely linked. From top view of MSC interdigital electrodes, Fig. 2d reveals that the MSC electrodes have a clear edge etched by laser. The width and length of the electrode are 369 μm and 3.8 mm, respectively. The gap between interdigital electrodes is 310 μm. Figure 2e is the enlarged view of interdigital electrodes, showing a regular shape. Further magnified picture of the interdigital electrode (Fig. 2f) demonstrates a flat surface with a little wrinkle, indicating that the PEDOT:PSS and [EMIM][TFSI] are well binding to form a robust structure.

Strong interactions between PEDOT:PSS and [EMIM][TFSI] are further shown in Fig. 2g-i. The vibrational modes of PEDOT are located at 1504, 1436, and 1368 cm^−1^, assigned to the *C*_α_ = *C*_β_ asymmetrical, *C*_α_ = *C*_β_ symmetrical, *C*_β_ − *C*_β_ stretching vibrations, respectively (Fig. 2g) [21]. The band in the spectrum of PE slightly redshifted to 1432 cm^−1^, reflecting that the average conjugation length of PEDOT chains was elongated because of the change of their conformation from a coiled benzene structure (coil) to a linear quinoid structure [18, 21, 22], which leads to an improvement of IWC-MSC conductive layer. The peak at 1566 cm^−1^ ascribes to PSS bond stretching and bending vibrations. XRD pattern in Fig. 2h shows that two well-defined peaks at 18.4° and 25.7° correspond to the amorphous halo of PSS and the interchain planar π–π stacking distance *d*_010_ of PEDOT, respectively (related to the lattice spacings *d* = 4.7 and 3.5 Å, according to Bragg's law) [23]. The peak at 12.1° significantly increased after mixed with [EMIM][TFSI], indicating that the increased lamella stacking and improved crystallinity of PE conductive layer, thus enhancing the conductivity of PE electrodes and wireless charging coil [24]. Main peaks of PEDOT:PSS in the Fourier transform infrared spectrometer (FTIR) spectrum (Fig. 2i) are located at 1042, 1012 (–SO_3_), 1265, 1129 and 1064 cm^−1^ (C–O). Two new peaks, 1345 and 1328 cm^−1^, are attributed to –SO_2_ stretching and C–SO_2_–N bending of TFSI anions [23], respectively, which verified the existence and bonding of [EMIM][TFSI] in PE conductive layer. Energy-dispersive spectrum (EDS) has also been carried out (Fig. S3). It is obvious that the F atoms have increased significantly after adding [EMIM][TFSI] into PEDOT:PSS of PE electrodes of MSC. Besides, element mappings of PE electrodes of MSC further display distribution of C, N, O, F and S atoms (Fig. S4), indicating that the [EMIM][TFSI] has mixed homogeneously with PEDOT:PSS in PE electrodes.

### Electrochemical Performances Measurement of MSC

**Fig. 3:**
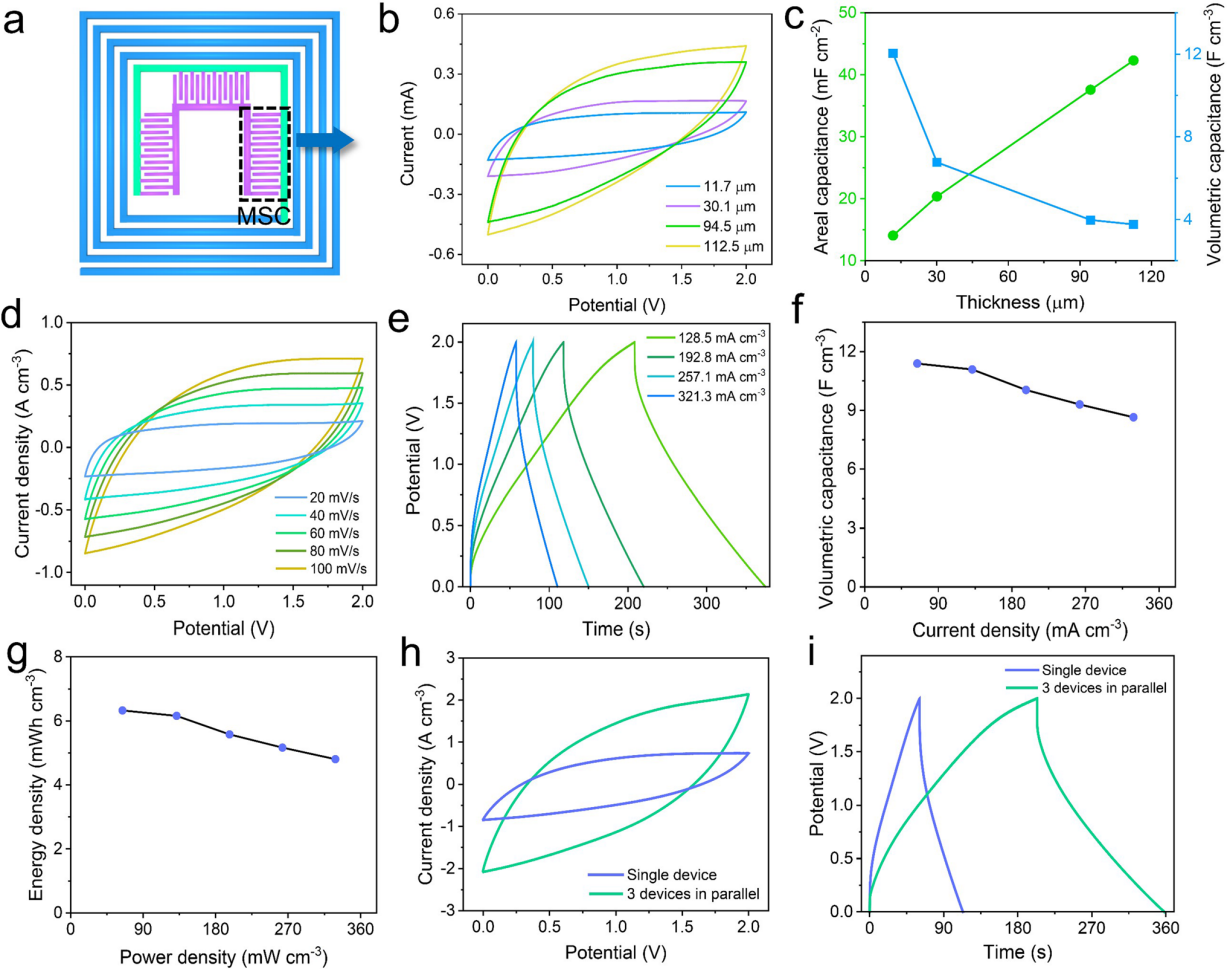
Electrochemical properties of MSC in the IWC-MSC. **a** Scheme of one MSC in the integrated IWC-MSC. **b** CV curves measured at scan rates of 20–100 mV s^−1^. **c** Galvanostatic charging/discharging curves of a single MSC with different current densities. **d** IR_drop_ related to MSC internal resistance vs different discharge current densities. **e** Nyquist plot of a single MSC unit; The inset is magnified details of the Nyquist plot. **f** The specific capacitance of a single MSC calculated from the discharging time under different currents. **g** Power and energy density of MSC. **h** CV curves of MSCs connected in parallel. **i** Galvanostatic charging/discharging curves of a group of three MSCs connected in parallel

Electrochemical performances of MSC were measured. The IWC-MSC contains three parallel MSCs, and single MSC is chosen to test (Fig. 3a). In Fig. 3b, it is obvious to see that the enclosed area of cyclic voltammetry (CV) curves increases with the growth of IWC-MSC thickness. This is because the mass of the electrochemical activated materials PEDOT:PSS/[EMIM][TFSI] increases and the MSC capacitance improves. Benefitting from the high operating voltage of ionic liquid electrolyte, the MSC potential window widens to 2 V, larger than most of aqueous electrolyte-based MSC. Areal and volumetric capacitance are also calculated according to CV curves (Fig. 3c). With the thickness improvement, the areal capacitance gradually increases to 42.3 mF cm^−2^, but the volumetric capacitance decreases on the contrary. To satisfy the practical requirement for ultrathin electronic skin, the thinnest thickness (11.7 μm) of MSC electrode is selected to investigate the electrochemical performance (Fig. 3d-i). Figure 3d displays the CV curves of single MSC at different scanning rates ranging from 20 to 100 mV s^−1^. All of the CV curves demonstrate near rectangular shape, revealing that the electrical double layers are formed within the thin film MSC electrodes and solid electrolyte interface. Little deviation from rectangular shape of the CV curves is attributed by the overlapping effect of double-layer and pseudocapacitive charge storage mechanisms [25]. Galvanostatic charge–discharge (GCD) curves are given in Fig. 3e. The curves are near-triangle shape, confirming the dominant electric double-layer energy storage mechanism of MSC. The IR_drop_ at the beginning of discharging line is quite small and demonstrates a linear relationship with the current density (from 64.3 to 328.7 mA cm^−3^) (Fig. S5), verifying low internal resistance of MSC device [26]. Figure 3f reveals the volumetric capacitance of the single MSC calculated according to the current density. The highest volumetric capacitance is achieved to 11.39 F cm^−3^ at the current density of 64.3 mA cm^−3^ (Fig. 3f). At a high current density of 328.7 mA cm^−3^, the volumetric capacitance also has a high value of 8.6 F cm^−3^, which is higher than most of micro-supercapacitors (Fig. S6) [26–32]. Capacitance of PEDOT:PSS comes from the double-layer charges on the interface between PEDOT-rich and PSS-rich grains [33]. According to the capacitance in Fig. 3f, the energy and power density of MSC are obtained (Fig. 3g). It exhibits a high energy density of 6.3 mWh cm^−3^ at the power density of 64.3 mW cm^−3^, and 4.8 mWh cm^−3^ at the power density of 328.7 mW cm^−3^, which is superior to most of micro-supercapacitors (Fig. S7) [32, 34–38]. As obviously shown in Fig. S8, the MSC with PE electrodes enabled with larger capacitance than the MSC only with PEDOT:PSS electrodes. It is confirmed that the excellent capacitive behavior of PE electrodes comes from its high conductivity, which is largely improved by [EMIM][TFSI] addition. The increase in electrical conductivity is partially due to a selective removal of PSS over PEDOT. It is well known that the PEDOT:PSS shows poor electrical transport properties, which caused by excessive insulating hydrophilic PSS chains encapsulating the conductive hydrophobic PEDOT cores, and inhibits PEDOT conducting networks formation [39]. Consequently, the treatment with ionic liquids on PEDOT:PSS is to exchange PSS to [TFSI]^−^ counterions and induce PEDOT chains fibrillar structure thus shortening the π–π interchain distances. As a result, the delocalization of π-electrons over the PEDOT conjugated polymer backbone brings faster charge carrier mobility, improving the conductivity of PEDOT:PSS [18, 19]. This is verified by X-ray photoelectron spectroscopy (XPS) spectrum in Fig. S9, which shows sulfur (S) 2*p* peaks of PEDOT:PSS and PEDOT:PSS/[EMIM][TFSI] of PE electrode. S atoms of thiophene in PEDOT and of sulfonate in PSS have different binding energies: the lower energy peaks (164.6 and 163.4 eV) correspond to the S atoms in PEDOT and the higher energy peaks (169 and 167.8 eV) correspond to the PSS, respectively. It is obvious to see that the PSS energy peaks of PEDOT:PSS shift to lower energy in PEDOT:PSS/[EMIM][TFSI], demonstrating weaker interactions between PEDOT and PSS [40].

To investigate the equivalent series resistance (ESR) and charge transfer mechanism, impedance spectroscopy is conducted. As shown in Fig. S10, the near semicircle in the high-frequency region shows low charge transfer resistance, and the steep line at low-frequency region indicates a good ion diffusion. The interception of X axis (Fig. S10 inset) delivers an ESR value of 403.2 Ω, which means the internal resistance of MSC is a little high, owing to the all-solid-state building materials. Additionally, the cycling stability of charging and discharging is also demonstrated. The capacitance remains 84.15% of the initial capacity after 3000 cycles (Fig. S11a), and the Coulombic efficiency changes to 47.54% from 48.09% of the initial state (Fig. S11b). Reduction of capacitance and low Coulombic efficiency may attribute to the electrostatic interaction between [TFSI]^−^ of electrolyte and PEDOT^+^ of electrodes [20], resulting in ions decrease in the electrolyte, thus bringing down the capacitance and Coulombic efficiency of MSC. To meet the practical energy requirement, three MSCs can be connected in parallel presenting three times closed area of the single MSC in CV curves (Fig. 3h) and providing three times for discharging of single MSC (Fig. 3i).

### Wireless Charging Performance Measurement of WCC

**Fig. 4:**
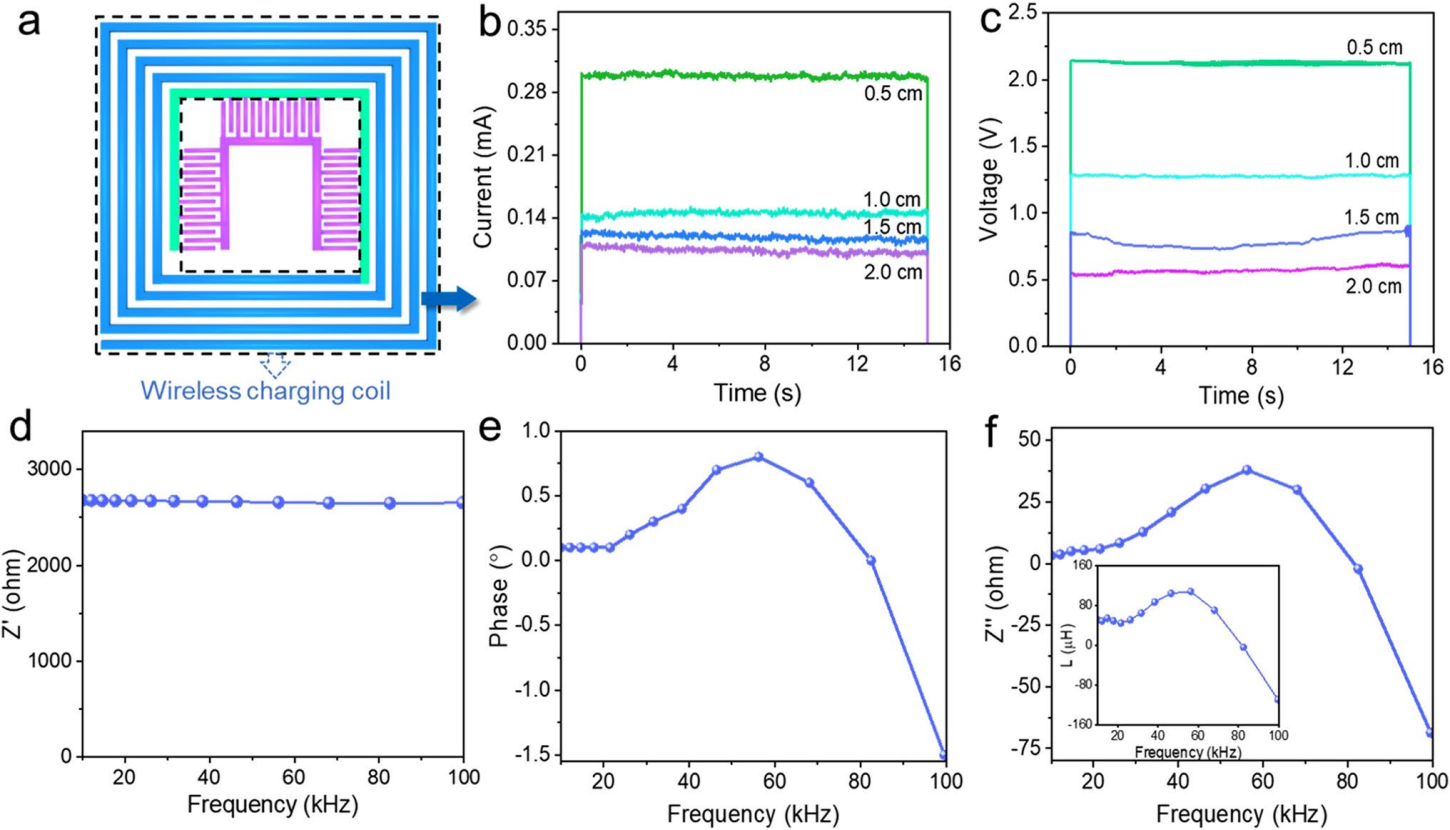
Performance characterization of wireless charging coil of IWC-MSC. **a** Scheme of WCC in IWC-MSC. **b** The received current, and **c** voltage of WCC of IWC-MSC under different distance to wireless transmitter during wireless charging process. **d** Real impedance, **e** phase and **f** calculated inductance and imaginary impedance (inset) of WCC

Inductance performance of PE wireless charging coil is investigated. Place the IWC-MSC near the wireless transmitter, where the WCC of IWC-MSC is used as a wireless receiver to charge MSC. The distance between the receiver and transmitter ranges from 0.5 to 2.0 cm. With the distance increasing, the received current and voltage increase accordingly (Fig. 4b, c). The highest current and voltage are 0.3 mA and 2.1 V respectively, demonstrating a high inductance performance of WCC. Subsequently, wireless charging coil frequency was measured from 10 to 100 kHz, and the obtained real impedance, phase, imaginary impedance and inductance are displayed in Fig. 4d–f, respectively. In Fig. 4d, the real impedance almost has no variation within the whole frequency, which means the PE WCC resistance (about 2.7 kΩ) and direct current loss are stable within the measurement range without causing fluctuations in transmission efficiency. Compared with PE WCC, the commercial antennas are usually made of metal, whose free electrons have skin effect. When the frequency gets higher, the free electrons tend to accumulate together, resulting in a lower conductivity. The inductance of the WCC in Fig. 4f inset was obtained according to the imaginary impedance in Fig. 4f by using formula:

4 $$L\left( {\text{H}} \right) = \frac{{Z^{{{\prime \prime }}} \left( \Omega \right)}}{{2\pi f\, \left( {{\text{Hz}}} \right)}}$$

where *L* is the inductance of the WCC; *Z*″ is the imaginary impedance of the WCC; and *f* is the measured frequency.

Besides, by comparing Fig. 4d, e, both of the imaginary impedance and phase have experienced zero near 90 kHz, so it can be inferred that the resonant frequency of the coil is 90 kHz.

### Performance and Application of IWC-MSC

**Fig. 5:**
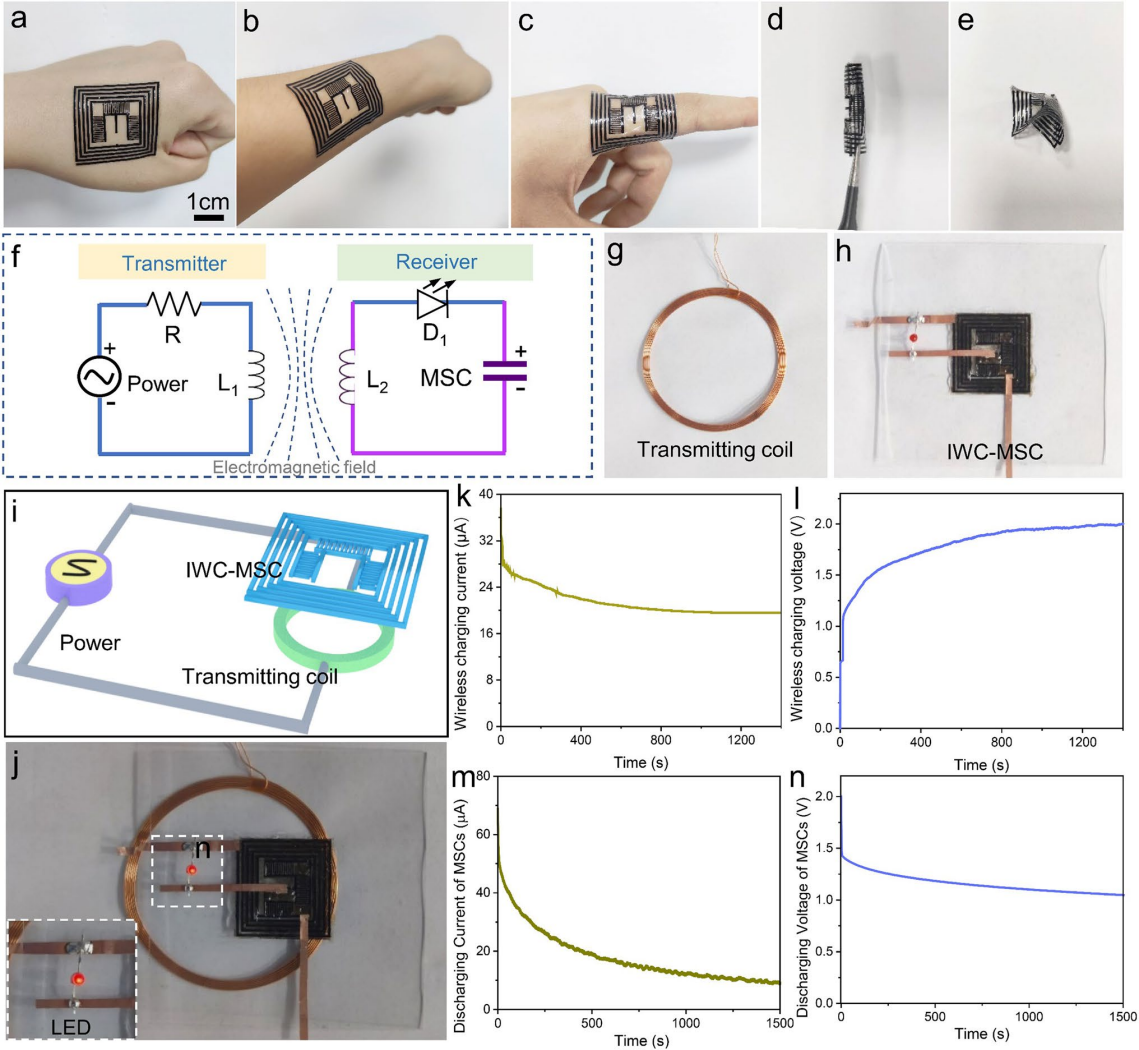
Display and application of IWC-MSC. **a** Image of IWC-MSC attaching conformably on the human fist, **b** arm and **c** finger. Pictures of **d** rolled and **e** crumpled IWC-MSC. **f** Circuit and **i** schematic diagram of the wireless charging system. The purple part is IWC-MSC. Photographs of **g** the wireless transmitting coil of wireless transmitter and **h** the wireless charging receiver. **j** A picture of wireless charging system during wireless charging. The inset is red LED lighting during wireless charging. **k** Induced current and **l** voltage of WCC during wireless charging process. **m** Discharging current curve and **n** discharging voltage curve of MSC after wireless charging

The appearance of IWC-MSC is recorded. Owing to the flexible and soft property of IWC-MSC, the IWC-MSC can be attached conformably with curved surface of human fist (Fig. 5a), arm (Fig. 5b) and finger (Fig. 5c), showing shape-adjustment, thin and transparent appearance, which are excellent advantages to be used as electronic skin. The mass of the whole device is 166 ± 4 mg, demonstrating enough lightweight to be used as electronic skin power source. Besides, benefitting from the tight and all-in-one structure, the IWC-MSC can also tolerant large deformation, such as rolling and crumpling (Fig. 5d, e and Movie S1), without influencing the device performance. It is approved that the performance of MSC is stable after bending for different angles under 1.9 mm bending radius (Fig. S12).

For practical application, the IWC-MSC was assembled with a red LED to reveal the wireless charging process vividly. IWC-MSC (purple parts) and the red LED are used as the wireless charging receiver, as shown in Fig. 5h, assembled by copper foil. The constructed wireless charging system circuit diagram and schematic diagram are illustrated in Fig. 5f-i. As displayed in Fig. 5g, the transmitting coil of wireless transmitter is an 8-cm-diameter circle, made of copper wires. The IWC-MSC is fixed on a glass plate to support the soft and thin device, and flatten the wireless charging coil for maximum wireless charging effects. During wireless charging process, the IWC-MSC receiver (Fig. 5h) is placed above the transmitting coil at a distance of 0.5 cm. While wireless charging, the red LED lights up, due to the wireless charging energy transference from transmitter to receiver (Fig. 5j), indicating that the wireless charging energy is high enough to light a LED.

The wireless charging and stored energy of IWC-MSC were also measured. The wireless charging current and voltage of WCC in IWC-MSC are illustrated in Fig. 5k, l. It is obvious to see that the current gradually decreased from 37.5 to 19.6 μA with the wireless charging process going (Fig. 5k). This is resulted by the increased resistance of MSC. Opposite charges gradually accumulated at the interface of MSC electrode and electrolyte, improving the repulsion for homologous charges, which presents increasing resistance of MSC. Similarly, the slope of wireless charging voltage curve declines as charging time goes by, confirming the increased resistance of MSC (Fig. 5l). Voltage of MSC finally reached to 2 V after 1400 s wireless charging.

To test the energy storage performance of MSC of IWC-MSC, discharging current and voltage were tested. After wireless charging for 1400 s, the initial discharging current of MSC is up to 68.8 μA and declines to 8.9 μA slowly after 1500 s discharging time (Fig. 5m). Besides, the released voltage is 2 V at the beginning of discharge (Fig. 5n), but quickly deduces to 1.5 V because of the high inert resistance of MSC, which may be further improved by other high conductive electrode materials. The calculated driving power is 137.6 μW, showing great potential in driving low-power electronics.

## Conclusion

In conclusion, we developed a skin-like ultracompatible all-in-one wireless charging micro-supercapacitor skin, which electrolyte, electrode and substrate are all evaporated by solution precursor. This preparation materials and method make the integrated device a tight and compact structure, significantly beneficial to attach with curving and crinkle human skin. Besides, the thickness of IWC-MSC electrodes can be regulated varying from 11.7 to 112.5 μm, showing strong adaptability for application. Moreover, the IWC-MSC device could be wireless charged to store electricity into MSC with a high capacity of 11.39 F cm^−3^, indicating a great potential as an autonomous power source for electronic skin and compatible wearables. This work not only presents a new method to build thin and compatible skin-like power source, but also promotes the convenience for energy supply in untouchable human organs.

## Supplementary Information

Below is the link to the electronic supplementary material.

Supplementary file1 (MP4 33410 KB)

Supplementary file2 (PDF 770 KB)
