# Supplementary material for: Ultraconformable Integrated Wireless Charging Micro-Supercapacitor Skin
Source: Nanomicro Lett. 2024 Feb 19;16:123. doi: 10.1007/s40820-024-01352-1 (PMC10876509; doi:10.1007/s40820-024-01352-1)
Supplement: Supplementary file 2 — Supplementary file2 (PDF 770 KB) [file 40820_2024_1352_MOESM2_ESM.pdf]

Supporting Information for

Ultraconformable Integrated Wireless Charging Micro-Supercapacitor Skin

Chang Gao[1,*], Qing You[1], Jiancheng Huang[2], Jingye Sun[1], Xuan Yao[1], Mingqiang Zhu[1], Yang Zhao[3], Tao Deng[1,*]

[1]School of Electronic and Information Engineering, Beijing Jiaotong University, Beijing 100044, P. R. China

[2]School of Microelectronics, Tianjin University, Tianjin 300072, P. R. China

[3]Key Laboratory of Cluster Science Ministry of Education of China, Beijing Key Laboratory of Photoelectronic/Electrophotonic Conversion Materials, School of Chemistry and Chemical Engineering, Beijing Institute of Technology, Beijing 100081, P. R. China

*Corresponding authors. E-mail: changgao@bjtu.edu.cn (Chang Gao); dengtao@bjtu.edu.cn (Tao Deng)

Supplementary Figures

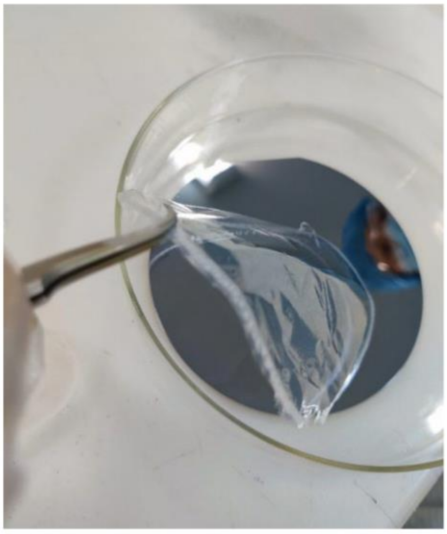

Fig. S1 PVDF-HFP substrate of IWC-MSC

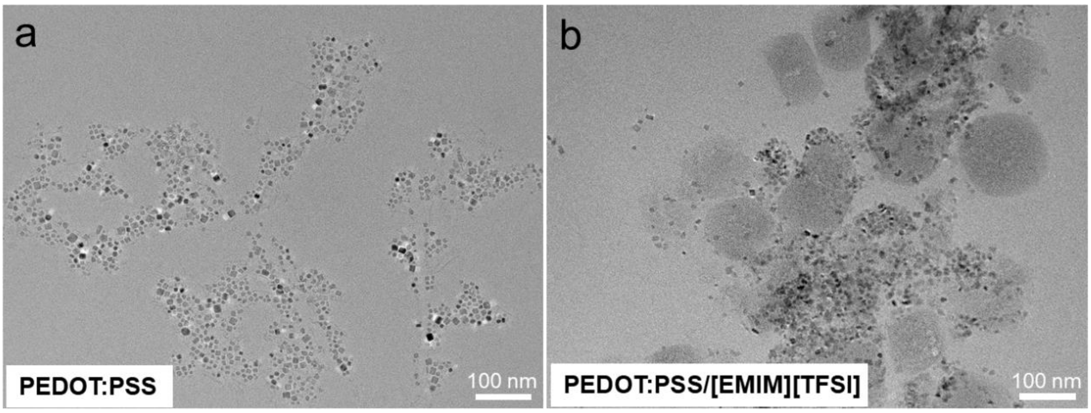

Fig. S2 TEM images of (a) PEDOT:PSS and (b) PEDOT:PSS/[EMIM][TFSI] electrode materials

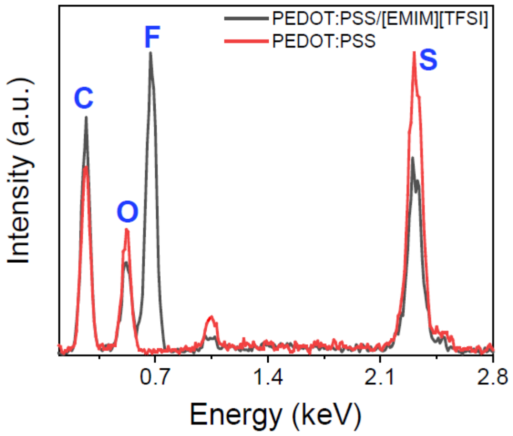

Fig. S3 EDS image of MSC PE electrode and PEDOT:PSS

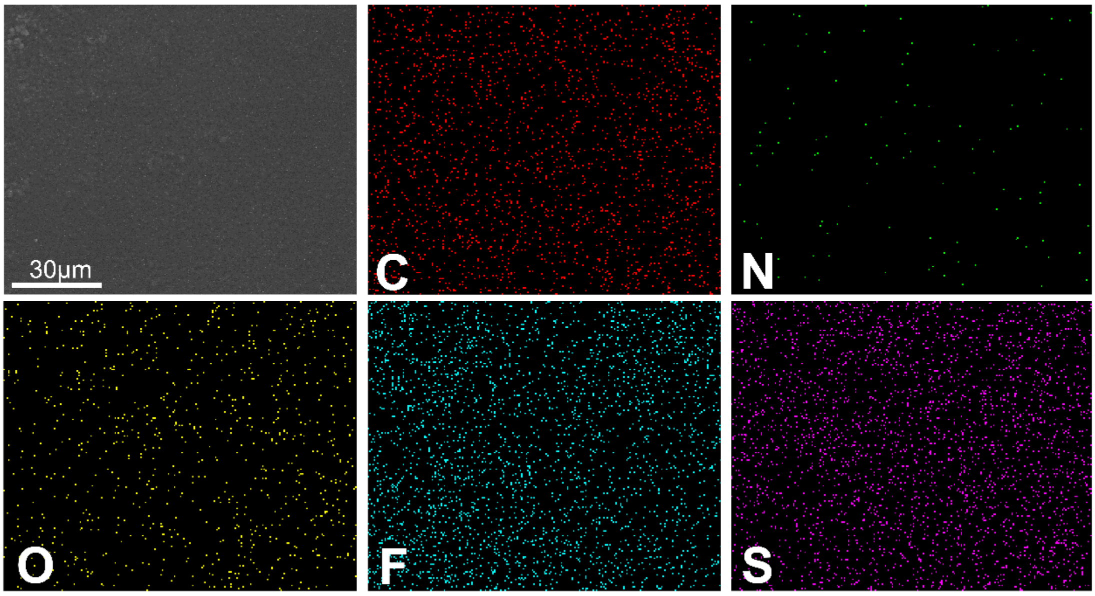

Fig. S4 Element mappings for C, N, O, F and S atoms in PE electrodes of MSC

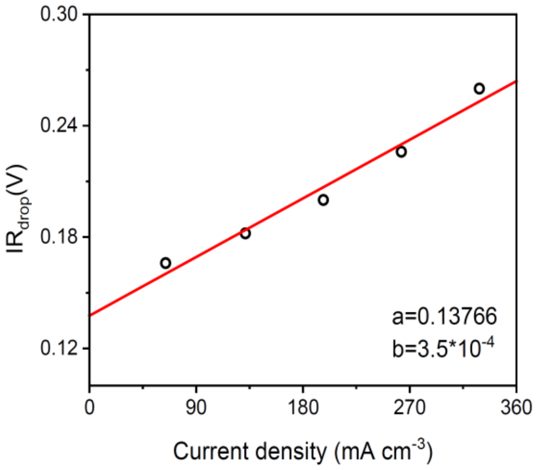

Fig. S5 IR_{drop} at the initial state of discharging curves under different current densities

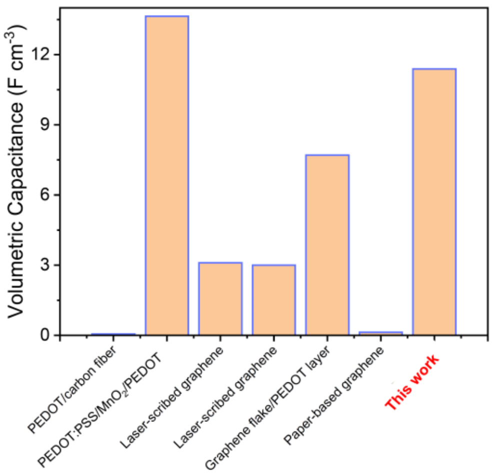

Fig. S6 The comparison of volumetric capacitance of single MSC and other reported micro-supercapacitors [S1-S7]

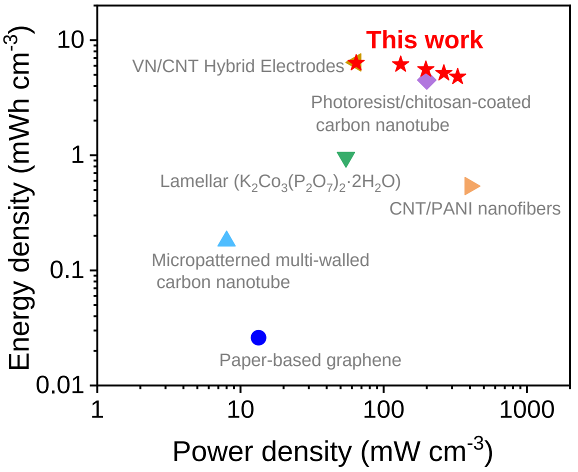

Fig. S7 Ragone plots of the prepared single MSC compared with other micro-supercapacitors

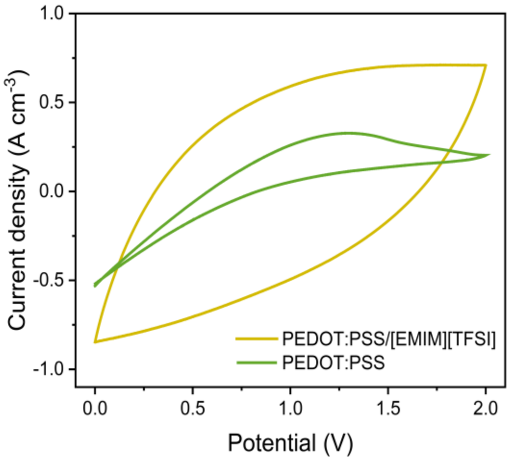

Fig. S8 Comparison of CV curves of PE MSC and PEDOT:PSS MSC

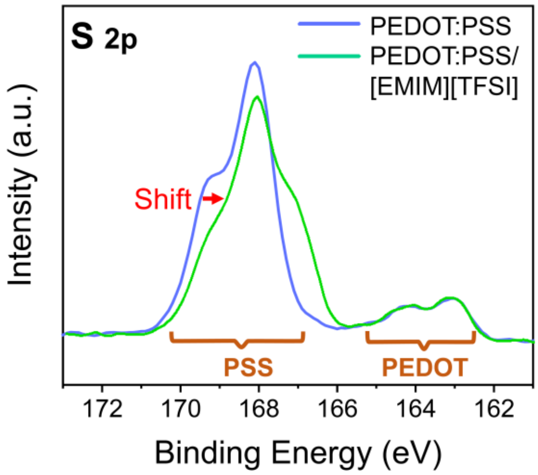

Fig. S9 (**a**) XPS images and (**b**) TEM pictures of PEDOT:PSS and PEDOT:PSS/[EMIM][TFSI] mixture of PE materials

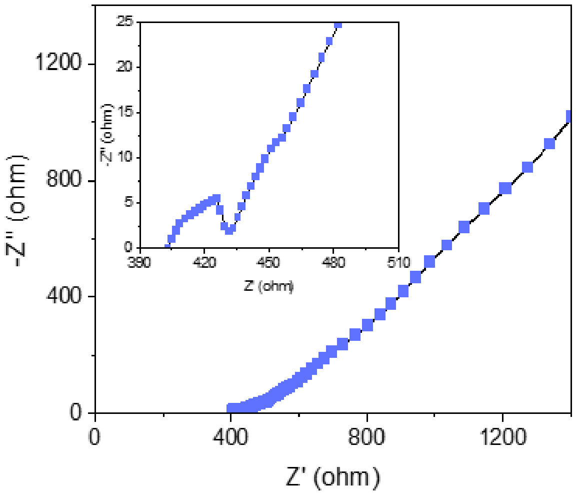

Fig. S10 The Nyquist plot of single MSC. The inset is the enlarge plot of X axis

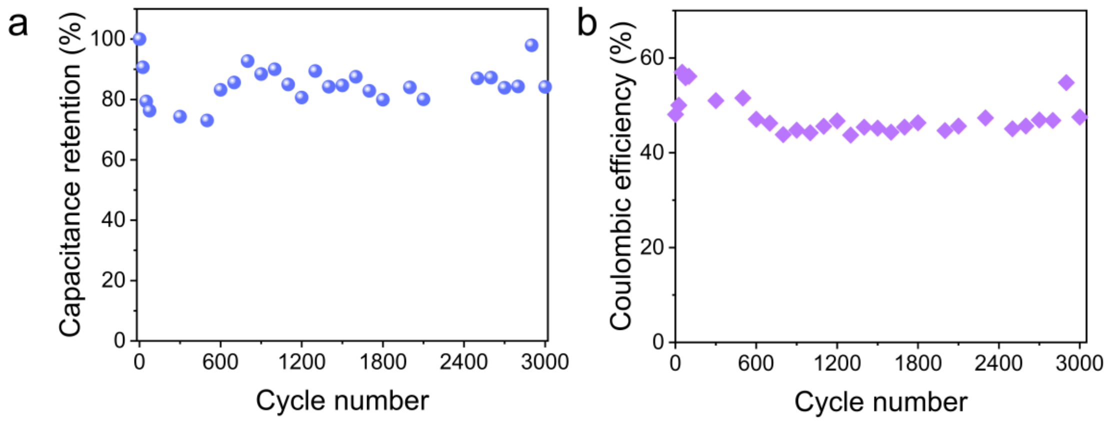

Fig. S11 (**a**) Cycling stability and (**b**) Coulombic efficiency of MSC after 3000 cycles of charging and discharging

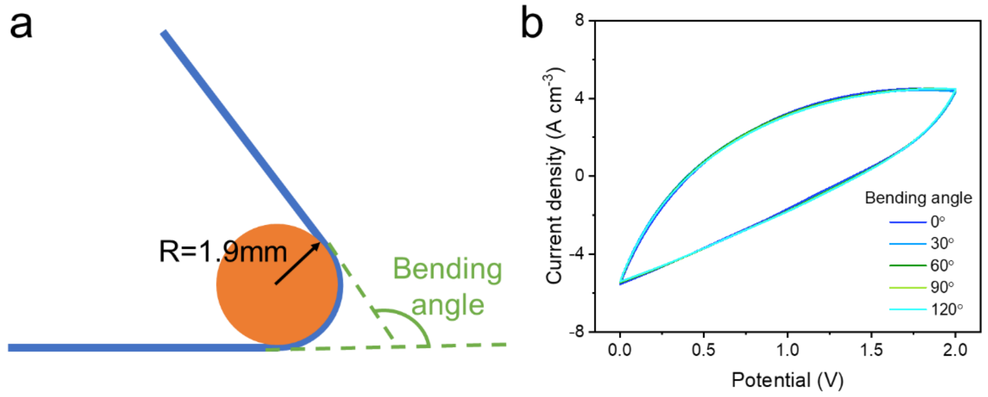

Fig. S12 Bending stability of MSC. (**a**) Scheme of bending angle and bending radius. (**b**) CV curves of MSC under different bending angles

Supplementary References

[S1] G. P. Pandey; A. C. Rastogi; C. R. Westgate, All-solid-state supercapacitors with poly(3,4-ethylenedioxythiophene)-coated carbon fiber paper electrodes and ionic liquid gel polymer electrolyte. J. Power Sources **245**, 857-865 (2014). https://doi.org/10.1016/j.jpowsour.2013.07.017

[S2] N. Kurra; M. K. Hota; H. N. Alshareef, Conducting polymer micro-supercapacitors for flexible energy storage and Ac line-filtering. Nano Energy **13**, 500-508 (2015). https://doi.org/10.1016/j.nanoen.2015.03.018

[S3] Y. Chen; J. Xu; Y. Yang; Y. Zhao; W. Yang, et al., The preparation and electrochemical properties of PEDOT:PSS/MnO_2/PEDOT ternary film and its application in flexible micro-supercapacitor. Electrochim. Acta **193**, 199-205 (2016). https://doi.org/10.1016/j.electacta.2016.02.021

[S4] W. Gao; N. Singh; L. Song; Z. Liu; A. L. M. Reddy, et al., Direct laser writing of

micro-supercapacitors on hydrated graphite oxide films. Nat. Nanotechnol. **6**(8), 496-500 (2011). https://doi.org/10.1038/nnano.2011.110

[S5] M. F. El-Kady; R. B. Kaner, Scalable fabrication of high-power graphene micro-supercapacitors for flexible and on-chip energy storage. Nat. Commun. **4**(2), 1475 (2013). https://doi.org/10.1038/ncomms2446

[S6] H. U. Lee; S. W. Kim, Pen lithography for flexible microsupercapacitors with layer-by-layer assembled graphene flake/PEDOT nanocomposite electrodes. J. Mater. Chem. A **5**(26), 13581-13590 (2017). https://doi.org/10.1039/c7ta02936e

[S7] B. Nagar; D. P. Dubal; L. Pires; A. Merkoçi; P. Gómez-Romero, Design and fabrication of printed paper-based hybrid micro-supercapacitor by using graphene and redox-active electrolyte. ChemSusChem **11**(11), 1849-1856 (2018). https://doi.org/10.1002/cssc.201800426
